# 624. Perceptions of Telemedicine among Infectious Disease Outpatient Providers during the COVID-19 Pandemic

**DOI:** 10.1093/ofid/ofab466.822

**Published:** 2021-12-04

**Authors:** Prishanya Pillai, Karen Carvajal, Angelina Winbush, Rohith Palli, Ted Louie

**Affiliations:** 1 Universitty of Rochester, Rochester, New York; 2 Division of Infectious Diseases, University of Rochester Medical Center, Rochester, New York; 3 School of Medicine and Dentistry, University of Rochester Medical Center, Rochester, New York; 4 University of Rochester, Rochester, New York

## Abstract

**Background:**

Access to infectious disease (ID) providers has been heavily impacted by COVID-19. Telemedicine (TM) has been viewed as a promising solution to the challenges in care delivery posed by this pandemic. However, perceptions of TM among ID providers remain unclear. This study investigated the impact of TM on outpatient ID during the COVID-19 pandemic, efficacy of TM in ID clinics, which clinical conditions within ID that TM may be best suited for, and influences on provider attitudes toward TM.

**Methods:**

We conducted online surveys of outpatient ID providers via the IDSA Idea Exchange and by reaching out to large ID private practices via email. Data was collected October 2020 - April 2021 and recorded through REDCap (Research and Electronic Data Capture). Associations were calculated using Hmisc in R and p-values adjusted using B-H. Missing values were imputed by median.

**Results:**

108 respondents completed the survey. 70.4% were attendings, 63% were under age 50, and 65.7% practiced in academic centers. 55.7% somewhat or strongly agreed there was a decline in outpatient visits. A median of 40% of outpatient visits during COVID were reported to be conducted via TM, divided equally between telephone and televideo (TV). 71.9% of providers somewhat or strongly agreed that TV visits were more effective than telephone visits. Reasons cited for TM as less effective than in person visits included lessened ability to examine the patient, diminished rapport, and inability to perform labwork. 85.2% somewhat or strongly agreed that patients responded favorably to TM. 89.8% of providers somewhat or strongly agreed they will continue to conduct TM visits when appropriate after COVID-19. Plans to continue TM after COVID were significantly associated with perceptions of patient response to TM. Respondents mentioned specific problems less suited to TM: new HIV patients, skin, soft tissue, and joint infections, fever, abdominal pain, transplant evaluations, rash, and wounds.

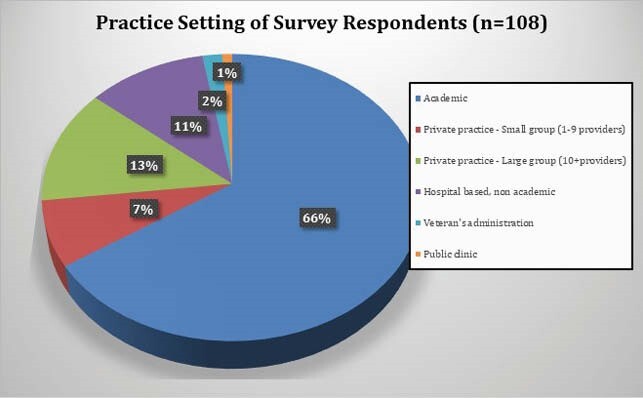

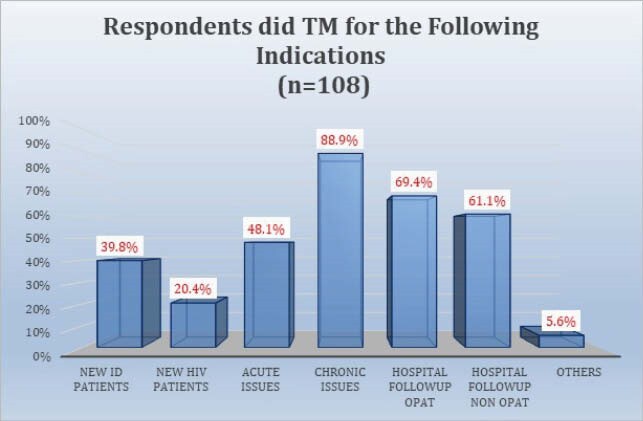

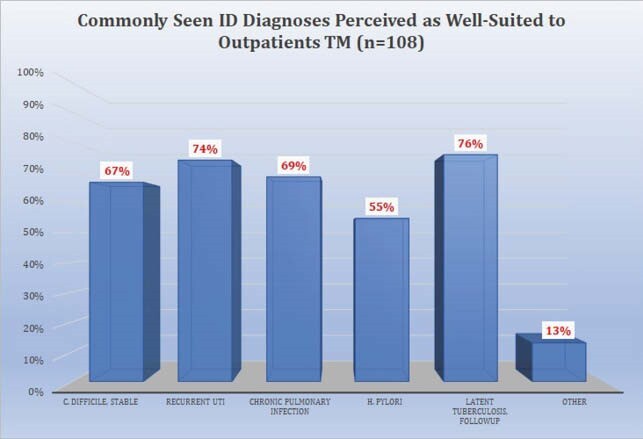

**Conclusion:**

The majority of providers felt their patients responded favorably to TM. Most providers plan to conduct TM visits after COVID-19. These plans are associated with views of patient response and comparability to other visit types. Specific diagnoses were cited as better suited for TM.

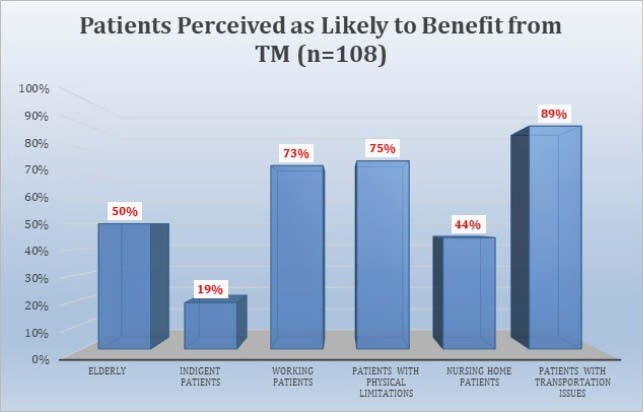

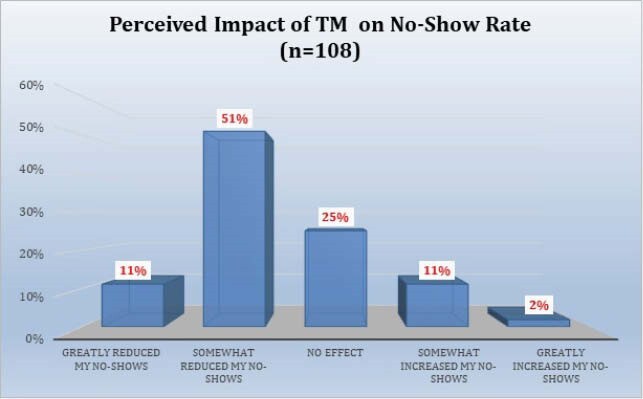

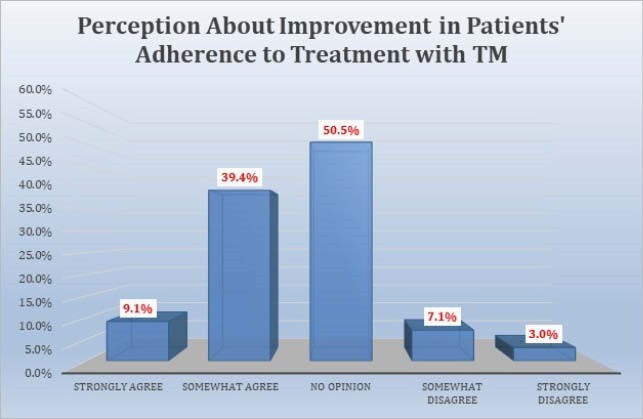

**Disclosures:**

**All Authors**: No reported disclosures

